# Against all odds: The road to success in the development of human immune reconstitution mice

**DOI:** 10.1002/ame2.12407

**Published:** 2024-04-09

**Authors:** Yixiao Bin, Jing Ren, Haowei Zhang, Tianjiao Zhang, Peijuan Liu, Zhiqian Xin, Haijiao Yang, Zhuan Feng, Zhinan Chen, Hai Zhang

**Affiliations:** ^1^ School of Basic Medical Sciences Shaanxi University of Chinese Medicine Xianyang China; ^2^ Department of Cell Biology, National Translational Science Center for Molecular Medicine Fourth Military Medical University Xi'an China; ^3^ State Key Laboratory of New Targets Discovery and Drug Development for Major Diseases Fourth Military Medical University Xi'an China; ^4^ Department of Occupational & Environmental Health and the Ministry of Education Key Lab of Hazard Assessment and Control in Special Operational Environment, School of Public Health Fourth Military Medical University Xi'an China

**Keywords:** hematopoietic stem cell, human immune reconstitution, immune response, immunodeficient mice, peripheral blood mononuclear cell, transplantation

## Abstract

The mouse genome has a high degree of homology with the human genome, and its physiological, biochemical, and developmental regulation mechanisms are similar to those of humans; therefore, mice are widely used as experimental animals. However, it is undeniable that interspecies differences between humans and mice can lead to experimental errors. The differences in the immune system have become an important factor limiting current immunological research. The application of immunodeficient mice provides a possible solution to these problems. By transplanting human immune cells or tissues, such as peripheral blood mononuclear cells or hematopoietic stem cells, into immunodeficient mice, a human immune system can be reconstituted in the mouse body, and the engrafted immune cells can elicit human‐specific immune responses. Researchers have been actively exploring the development and differentiation conditions of host recipient animals and grafts in order to achieve better immune reconstitution. Through genetic engineering methods, immunodeficient mice can be further modified to provide a favorable developmental and differentiation microenvironment for the grafts. From initially only being able to reconstruct single T lymphocyte lineages, it is now possible to reconstruct lymphoid and myeloid cells, providing important research tools for immunology‐related studies. In this review, we compare the differences in immune systems of humans and mice, describe the development history of human immune reconstitution from the perspectives of immunodeficient mice and grafts, and discuss the latest advances in enhancing the efficiency of human immune cell reconstitution, aiming to provide important references for immunological related researches.

## Graphical abstract

Over recent decades, researchers have been actively exploring the development and differentiation conditions of host recipient animals and grafts in order to achieve better immune reconstitution. The human immune system can be reconstituted in immunodeficient mice by transplanting human peripheral blood mononuclear cells or hematopoietic stem cells, and the engrafted immune cells can elicit human‐specific immune responses. At present, three different types of human immune reconstitution mice have been developed and human lymphoid and myeloid cell‐mediated immune systems have been reconstituted successfully in immunodeficient mice.

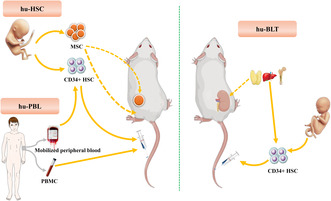

## 1 INTRODUCTION

The mouse has become the most widely used animal in biomedical research due to its small size, fast reproduction, well‐defined genetic background, and ease of manipulation. Over the last 100 years, thousands of different strains of inbred, outbred, hybrid, and mutant mice have been artificially bred for biomedical research^1^; however, due to the interspecies differences between humans and mice, the experimental results obtained in mice cannot be directly generalized to humans. Thus, it is necessary to develop a model organism that can closely simulate human pathological and physiological responses.

The immune response is an important mechanism for maintaining homeostasis in the body; however, for ethical reasons, the majority of experiments can only be conducted in mice, and the observations of immune responses in mice provide evidence for human immunological reactions. There are differences in immune cell composition, immune cell distribution, and immune molecule expression between humans and mice, thus making it difficult to deduce the overall picture of human immune responses based on results obtained in mice.^2^ For example, the first therapeutic antibody drug approved by the U.S. Food and Drug Administration, OKT3, did not induce cytokine storms in mice and monkeys during preclinical studies, but it caused typical cytokine storms in humans.^3^ Therefore, the artificial breeding of mice that fully exhibit human immune responses for biomedical research is difficult, and if mice are to be used for biomedical research, the experimental errors caused by interspecies differences must be overcome. Decades ago, researchers attempted to overcome these differences by introducing human genes into mice through transgenic methods or knocking out genes closely related to immune responses in mice; however, it has been shown that the knockout or introduction of single genes cannot completely eliminate these effects.^4^, ^5^, ^6^ Xenotransplantation is an effective method to address the experimental errors caused by interspecies differences.^7^ By selecting appropriate human immune tissues or cells and transplanting them into suitable mice, human immune tissues can be engrafted and functionally rebuilt in mice, thus overcoming the differences in immune responses between humans and mice.^8^ Researchers have made unremitting efforts over the last few decades, going through multiple stages of development and actively exploring the biological characteristics of donor grafts and host animals. They have successfully transplanted human peripheral blood mononuclear cells (PBMCs) and hematopoietic stem cells (HSCs) into different strains of immunodeficient mice, reconstructing the human immune system in mice. Starting from the initial reconstruction of the human lymphoid immune cells in the microenvironment of mice, it is now possible to reconstruct both lymphoid and myeloid immune cells, solving the current challenges in biomedical research and providing an ideal animal model for immunological research.

## 2 IMMUNE SYSTEM DIFFERENCES BETWEEN HUMANS AND MICE

Natural selection and artificial breeding are the mechanisms behind the differences in the immune systems of humans and mice. The last common ancestor of humans and mice lived approximately 65–75 million years ago.^9^ Comparative genomics studies have found that humans and mice share 80% of their genes, but only 40% of these genes are complete matches, with approximately 300 genes, including 169 immune‐related genes, showing species specificity and differences between the two species.^10^, ^11^

Humans and mice are mammals, and although there are no significant anatomical differences in the overall structure of their immune organs, there are differences in immune cell composition, immune cell distribution, and immune molecule expression.^2^ There is a significant difference in the percentage of lymphocytes and neutrophils in human and mouse peripheral blood. Human peripheral blood has a higher percentage of neutrophils and a lower percentage of lymphocytes, while the opposite is the case in mice.^12^ The immunoglobulin types in human and mouse sera are also different. In mice, IgG is divided into four subclasses, namely, IgG1, IgG2a, IgG2b, and IgG3, while in humans, IgG is divided into different subclasses, namely, IgG1, IgG2, IgG3, and IgG4. This difference leads to different binding abilities between different subclasses of IgG and Fc receptors.^13^ Although the anatomical structure of the spleen is similar in humans and mice, the distribution of T cells in the white pulp of the spleen differs. T cells in mice are clustered, while in humans, they are scattered in the germinal centers.^14^ There are also differences in the composition of immune cells in the epidermis of the skin between humans and mice. Dendritic epidermal γδ T cells (DETCs) are only found in the epidermis of mice, while humans have αβ T cells but no DETCs.^15^, ^16^ The interspecies differences between humans and mice also determine differences in immune molecule expression and function. Human neutrophils can express defense molecules with antimicrobial effects, while mouse neutrophils do not possess this function. In human neutrophils, FcαRI can mediate immune killing after binding with antibodies, but mouse neutrophils do not express FcαRI; instead, they use Fcα/μR to bind with IgM.^17^ Ly49 is an inhibitory receptor for NK and NKT cells in mice, while the inhibitory receptor for human NK cells is KIR, not Ly49.^18^ Similarly, there are differences in ligand binding between human and mouse NK cells. Human NKG2D can bind to its ligands MHC‐I and UL16, while mouse NKG2D binds to H‐60 and Rae1β to exert biological effects.^19^, ^20^ These interspecies differences in immune‐related genes also result in different functions. Mutation of the X‐linked interleukin 2 receptor subunit gamma chain gene (*IL2rg, CD132* or γc) affects the development of human T cells and NK cells, but not B cells; however, mutation of the *IL2rg* gene severely decreases the number of B cells in mice.^21^ Similarly, mutation of the *IL7R* gene inhibits the development of human T cells, but in mice, it affects both T and B cell development.^22^, ^23^ Interspecies differences between humans and mice also result in different immune response processes. In humans, activation of Th2 cells results in the recruitment of eosinophils and the production of IgE by B cells, which is the main defense mechanism against schistosomiasis; however, mice use Th1 cell‐produced IFN‐γ to combat parasite infection.^24^, ^25^ IFN‐αβ or IL‐4 combined with anti‐CD23 antibodies can induce human macrophages to produce inducible nitric oxide synthase (iNOS) to resist pathogenic microbial infections, while mouse macrophages can only produce significant levels of iNOS when induced by LPS and IFN‐γ.^26^, ^27^

The currently available laboratory mice are inbred or transgenic mice that have been artificially bred for a long time and are maintained in an SPF environment. They have lost genetic diversity and are sensitive to the external environment; moreover, the immune differences caused by interspecies differences further limit the range of application of these mice. However, the reconstruction of the human immune system in mice through xenotransplantation can effectively solve the problems encountered in mouse experiments.

## 3 CHOICE OF RECIPIENT HOST ANIMAL AND DONOR GRAFT

**FIGURE 1:**
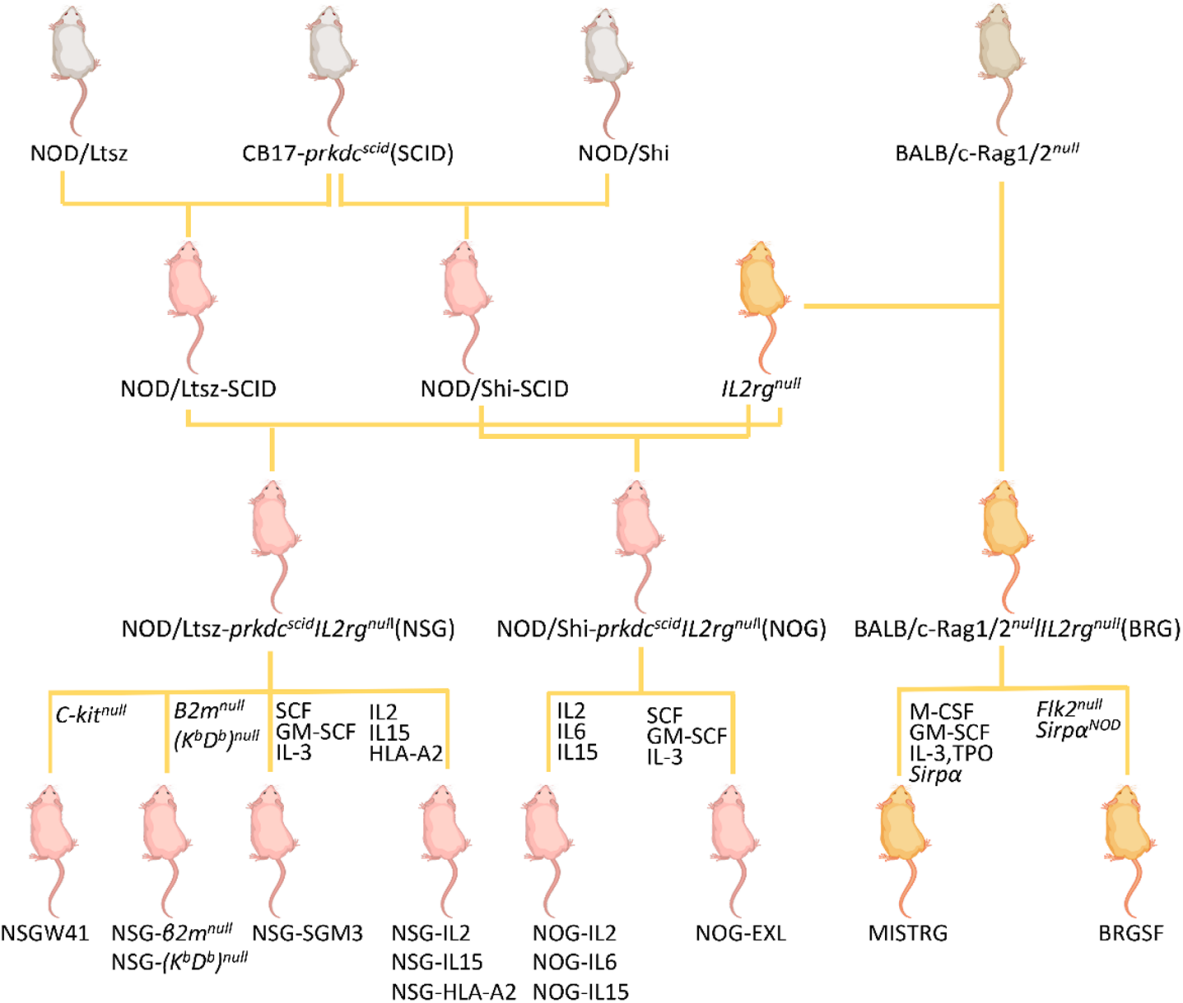
Diagram of immunodeficient mouse development.

Reconstructing the human immune system in mice through xenotransplantation involves both a donor graft and a recipient host animal. The donor graft from humans should include cells or tissues that are immunologically relevant, while the recipient host animal should be able to accept the donor graft and allow it to proliferate or differentiate in the body. The discovery and application of immunodeficient mice are important milestones in the process of human immune reconstruction. Donor grafts can survive in immunocompromised mice due to their immunodeficiency. The development of immunodeficient mice has progressed from the simplest types, such as nude mice (with T cell deficiency), to the currently used severe combined immunodeficient (SCID) mice, which have severe combined deficiencies in T, B, and NK cell functions. This development has gone through different stages (Figure 1). SCID mice now serve as a suitable host for human immune transplantation.

### 3.1 *prkdc* ^*−/−*^ mice

The discovery of hairless mice with a single T cell deficiency due to a *Foxn1* mutation paved the way for studying immunodeficient mice and provided favorable conditions for human xenotransplantation models.^28^, ^29^ The DNA‐dependent protein kinase (DNA‐PK), encoded by the *Prkdc* gene, is a critical component of the non‐homologous end joining pathway for repairing double‐strand breaks in DNA during V(D)J recombination. In addition to DNA‐PK, Artemis, Rag1/2, XRCC4 and Ligase IV are also important regulatory genes in the V(D)J recombination process. The complexes consisting of DNA‐PK/ Artemis, Rag1/2, and Ligase IV/XRCC4 complete the repair of double‐strand gaps during V(D)J recombination, generating diverse T and B cell receptors that mediate adaptive immune responses.^30^, ^31^, ^32^ Mutations in any of these genes in the complex result in abnormal V(D)J recombination, leading to impaired T and B cell development; this is also known as SCID.^33^ Introducing the mutated *Prkdc* gene in C.B‐17, C3H, and Beige mice can generate typical SCID animals.^34^, ^35^, ^36^ SCID mice exhibit more severe immune deficiencies than nude mice, making them a preferred model for immune reconstitution studies. Although SCID mice have combined T and B cell deficiencies, their NK cells, macrophages, and complement system function normally. Non‐obese diabetic (NOD) mice, on the other hand, have lower NK cell function, macrophage defects, and complement deficiency.^37^ NOD‐SCID mice were generated by crossing NOD mice with C.B‐17/SCID mice, which retained the characteristics of both strains with combined T and B cell deficiencies, as well as low NK cell function, macrophage defects, and complement deficiency^38^ (Figure 1). Human immune grafts develop and proliferate better in NOD‐SCID mice compared to SCID mice. The introduction of mutations in key genes involved in the repair of double‐strand gaps in V(D)J recombination, such as *Prkdc*, *Rag1/2*, *Dclre1c*, and *Ligase IV*, using embryonic stem cell targeting techniques, can also result in severe combined immunodeficiency SCID in mice with impaired T and B cell function.^39^, ^40^, ^41^, ^42^

### 3.2 *IL2rg* ^*−/−*^ mice

*IL2rg* encodes a glycoprotein mainly expressed on the surface of memory T cells and NK cells, serving as a common receptor subunit for various important immune factors such as IL‐2, IL‐4, IL‐7, IL‐9, IL‐15, and IL‐21. Janus kinase 3 (JAK3) is one of the crucial kinases that mediate IL‐2 receptor signaling, the interaction between IL2rg and JAK3 regulates the activity of immune cells, either a mutation in the *IL2rg* or *JAK3* gene can cause immune deficiency, characterized by NK cell deficiency, downregulation of cytokine expression, and inhibition of immune cell proliferation and differentiation.^43^, ^44^, ^45^, ^46^ *IL2rg* and *JAK3* mutations were introduced into NOD‐SCID mice in order to further enhance immune deficiency in the mice (Figure 1). The resulting NSG (NOD/LtSz‐Prkdc^scid^ Il2rg^tm1Wjl^/J), NOG (NOD/Shi‐Prkdc^scid^ Il2rg^tm1Sug^/Jic), and NOD/SCID‐JAK3^null^ mice not only retained the T and B cell deficiency, macrophage defects, and complement deficiency of NOD‐SCID mice, but also exhibited NK cell deficiency and downregulated expression of cytokines IL‐2, IL‐4, IL‐7, IL‐9, IL‐15, and IL‐21 due to the *IL2rg* mutation.^47^, ^48^ These mice are more immunodeficient than NOD‐SCID mice, *Rag1* or *Rag2* mutant mice, thus making them more suitable for human immune system engraftment. Using gene editing technologies such as ZFN, TALEN, and CRISPR/Cas9, severe combined immunodeficient mice with T, B, and NK cell defects, like NCG and BRG mice, can be generated by mutating *Prkdc*, *Rag1/2*, *Dclre1c*, and *IL2rg*.^49^, ^50^ Many different strains of SCID mice are currently used for human immune reconstitution, every strain has different biological characteristics, and the efficiency in engrafting human immune cells varies among strains. In general, NOD‐SCID mice with T and B cell defects have lower reconstitution efficiency than NSG, NOG, and BRG mice with combined T, B, and NK cell deficiencies, and the reconstitution effects in BRG mice are less pronounced than in NSG and NOG mice^51^, ^52^; therefore, NSG and NOG mice are currently the most widely used animal models for human immune reconstitution and immune system transplantation research.

### 3.3 Donor grafts

The thymus, bone marrow, lymph nodes, spleen, and mucosal tissues are the immune organs, where immune cells develop, differentiate, mature, and reside. Immune cells that have matured in the immune organs circulate through the bloodstream and play a part in the immune response; therefore, immune organs and peripheral blood have become the main choice for transplantation in the process of human immune reconstitution. In the early stages of human immune reconstitution, fetal thymus, bone marrow, lymph nodes, and other tissues were often transplanted,^53^, ^54^ but obtaining immune organs was difficult and the transplantation technique was demanding. Later, adult peripheral blood became a common choice for transplantation. PBMCs are a mixed population of mononuclear cells with a single nucleus found in peripheral blood, which includes T lymphocytes, B lymphocytes, NK cells, monocytes, phagocytes, and dendritic cells that are produced in the thymus, bone marrow, spleen, and other immune organs.^55^ The immune cells in PBMCs are mostly mature cells, and following transplantation, some of them can directly proliferate in the immunodeficient mice, thus partially reconstructing the human immune system.

CD34^+^ HSCs obtained from umbilical cord blood, the embryonic liver, mobilized peripheral blood, or bone marrow are precursors for all immune cells and possess the ability to self‐renew and differentiate into immune cells.^56^ Following transplantation, pluripotent CD34^+^ cells show different differentiation abilities, depending on the host animal, and can differentiate into different types of human immune cells for immune reconstitution.

## 4 HUMAN IMMUNE RECONSTITUTION MICE

**FIGURE 2:**
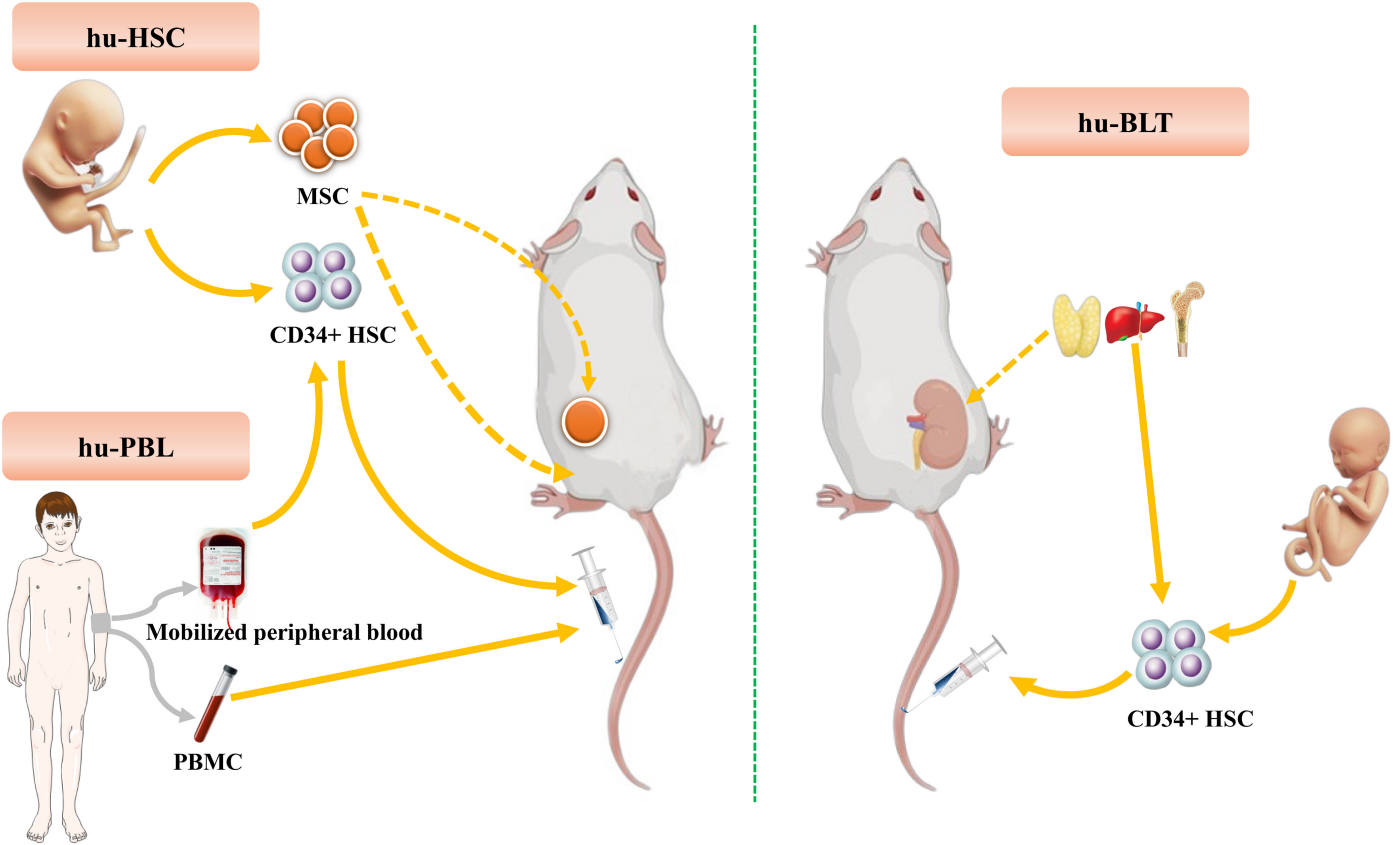
Diagram of human immune reconstitution mouse classification.

Humanized mice for immune reconstitution can be classified into three categories, namely, hu‐PBL, hu‐HSC, and hu‐BLT, based on the source of the graft and the host animal (Figure 2).

### 4.1 hu‐PBL immune reconstitution mice

Human PBMCs or human peripheral blood lymphocytes (hu‐PBLs) are easy to obtain and manipulate. They have already differentiated and matured in the human body, so they can be directly transplanted into immunodeficient mice for rapid reconstruction of human immune cells^57^ (Figure 2). This is a relatively simple and economical model, providing humanized mice with a human immune system. In the early stages, the PBMCs or lymphocytes were often transplanted into SCID mice, but with the discovery and application of NSG or NOG mice, which have higher degrees of immunodeficiency, these two types of mice are now commonly used for hu‐PBL reconstitution. The presence of human CD45^+^ T cells can be detected as early as 2 weeks after hu‐PBL transplantation. After 3 weeks, human CD45^+^CD3^+^ cells, including a large number of CD4^+^ and CD8^+^ T cell subsets, proliferate rapidly. Further analysis revealed an increase in the number of CD45RO^+^CD27^−^ effector T cells in the CD4^+^ and CD8^+^ T cell subsets, while the ratios of initial CD45RO^−^CD27^+^ T cells/CD45RO^+^CD27^+^ memory T cells and central CD45RO^+^CD27^+^CD62L^+^ memory T cells/CD45RO^+^CD27^+^CD62L^−^ effector memory T cells were both decreased. This indicates that in hu‐PBL‐SCID mice, the human T cell subset mainly comprises central memory T cells, while other T cell subsets such as FOXP3^+^CD25^+^CD127^low^ regulatory T cells are not found to proliferate.^58^, ^59^, ^60^ Human T cell subsets often colonize and proliferate in the peripheral blood, thymus, spleen, liver, and lymph nodes of mice, and the presence of different human T cell subsets can be detected in these tissues.^60^, ^61^ In hu‐PBL mice, only the proliferation of different types of human T cells can be detected, while CD19^+^ B cells can only be maintained at very low levels for a short time in the bone marrow and spleen of these mice.^62^ Human NK cells and other myeloid immune cells, such as macrophages, dendritic cells, and granulocytes, cannot proliferate in the body of hu‐PBL mice.^63^ Due to the differences in human and mouse MHC antigens, the transplantation of human PBMCs often causes graft‐versus‐host disease (GVHD) in hu‐PBL mice in the short term, leading to weight loss, a hunching posture, hair loss, and decreased activity; GVHD can even result in death, thus shortening the experimental window.^64^, ^65^, ^66^ Therefore, hu‐PBL mice are only used to observe T cell‐mediated immune responses and conduct short‐term experimental studies.

### 4.2 hu‐HSC immune reconstitution mice

CD34^+^ HSCs have the ability to differentiate into multiple lineages, and transplantation into immunocompromised mice with the help of the animal's microenvironment has become an important method for human immune reconstruction (Figure 2). HSCs used for transplantation are typically sourced from fetal liver, umbilical cord blood, bone marrow, or G‐CSF‐mobilized peripheral blood.^67^, ^68^ Notably, the source of HSCs can influence the function of human T cells that develop in the transplanted mice. T cells derived from CD34^+^ HSCs from fetal sources have higher immunotolerance compared to those from adult sources.^69^ Following transplantation into NSG or NOG mice, HSCs can partially reconstitute the human immune system within 10–12 weeks. Human innate immune response cells, adaptive immune response cells, and a small number of red blood cells and platelets can be detected in the mice.^70^, ^71^, ^72^ Unlike PBMC transplantation, which only allows the reconstitution of human T cells, in hu‐HSC mice, various types of human immune cells can be detected. In addition, HSCs from CD34^+^ HSC transplants can differentiate into different lineages of immune cells in SCID mice, leading to a lower rejection response from the mouse immune system and delayed development of GVHD^71^; however, hu‐HSC mice are not an ideal animal model for immune reconstruction, as human myeloid cells, such as macrophages, dendritic cells, and NK cells do not develop fully in the mice. This results in decreased production of antigen‐specific IgGs and reduced HLA restriction. In light of these characteristics, hu‐HSC mice are suitable for conducting experiments that require long research periods and observation of various immune responses.

### 4.3 hu‐BLT immune reconstitution mice

T cell development depends on the thymic microenvironment, but immunodeficient mice have severe thymus degeneration, which is one of the reasons for the low efficiency of immune reconstruction. Transplantation of fetal thymic and liver tissues into immunodeficient mice under the renal capsule creates an artificial thymic microenvironment, which overcomes the problem of impaired T cell development due to thymus degeneration in mice^73^ (Figure 2). Based on this principle, researchers have created BLT mice to improve immune reconstruction efficiency. BLT mice are created by simultaneous transplantation of fetal thymic and liver tissues into NSG or NOG mice under the renal capsule, followed by injection of CD34^+^ HSCs to reconstitute the human immune system. The humanized thymus in BLT mice provides an ideal environment for T cell development, education, and selection, resulting in HLA‐restricted T cells. Further, this mouse model can reconstitute human myeloid cells, including monocytes, granulocytes, and the mucosal immune system.^74^, ^75^

Transplanted human tissues in recipient mice establish a complete human hematopoietic microenvironment, enhancing the multi‐lineage reconstitution of human hematopoietic cells. Notably, a functional and complete human immune system, including T cells, B cells, and dendritic cells, is developed. These mice also produce high levels of human IgM and IgG antibodies. The development of T cells relies on selection in the human thymus. BLT mice, which contain diverse HLA‐restricted T cells, can establish effective adaptive immune responses; therefore, BLT mice are often used in research related to adaptive immune responses, such as HIV infection.^76^ The use of BLT mouse models is limited by the availability of human embryonic tissues, and due to the positive selection in the thymus being specific to human T cells, T cells with mouse MHC specificity cannot be eliminated. As a result, hu‐BLT‐SCID mice are more prone to developing GVHD than other humanized mouse models.^77^

## 5 OPTIMIZATION OF HUMAN IMMUNE RECONSTITUTION EFFECT

### 5.1 Improvements in immunodeficient mice

#### 5.1.1 Promoting multidirectional differentiation of HSCs

HSCs are a type of progenitor cell that can self‐renew, differentiate, and regenerate into various types of blood cells. The regulation, proliferation, and differentiation of HSCs involve a series of hematopoietic growth factors. Positive regulators such as stem cell factor (SCF), FLT3, colony‐stimulating factors (CSFs), IL‐2, IL‐3, IL‐6, IL‐15, and thrombopoietin (TPO) promote HSC differentiation, while negative regulators such as TGF‐β, TNF‐α, β, and chemokines inhibit HSC differentiation.^78^, ^79^, ^80^ Positive regulators bind to receptors on HSCs, activating intracellular signaling pathways such as the ERK, JNK, and TKR pathways, inducing the division and proliferation of hematopoietic progenitor cells, and promoting the transition of HSCs from the G0 phase to the G1 phase, accelerating HSC proliferation and differentiation.^81^, ^82^ Negative regulators such as TGF‐β induce HSCs to enter the G0 phase, inhibiting HSC proliferation and differentiation.^83^ Under the influence of positive regulators, HSCs can differentiate into various blood cell lineages, including myeloid cells (monocytes, macrophages, granulocytes, red blood cells, megakaryocytes/platelets, and dendritic cells) and lymphoid cells (T, B, and NK cells).

As mentioned above, following the transplantation of human HSCs into immunodeficient mice, the mice lack the microenvironment necessary for the proliferation and differentiation of human HSCs. Consequently, HSCs are unable to differentiate into lymphoid NK cells and myeloid cells such as granulocytes and macrophages; however, researchers have used genetic engineering methods to introduce positive regulatory factors that regulate HSC differentiation into the mice (Figure 1), thereby improving the developmental microenvironment for human HSCs and enabling their differentiation into various lineages of immune cells. IL‐2 is an important cytokine that induces the proliferation of NK cells; it activates various kinase pathways, increases NK cell activity, and promotes NK cell proliferation. IL‐15 upregulates the expression of surface receptors on NK cells and also promotes the expansion of CD56^+^ cells.^84^ By expressing human IL‐2 and IL‐15 in NOG or NSG mice, researchers have overcome the limitations of HSC differentiation into NK cells observed in prior studies. The differentiation and proliferation of CD56^+^ NK cells in the peripheral blood and spleen of these mice are more than 10 times higher than that in NOG mice. Further, these NK cells express various receptors, such as NKp30, NKp44, NKp46, NKG2D, and CD94, and produce cytotoxic factors such as perforin and granzyme upon stimulation.^84^, ^85^, ^86^

Granulocyte‐macrophage colony‐stimulating factor (GM‐CSF), SCF, IL‐3, and TPO are cytokines that are essential for the differentiation of CD34^+^ HSCs into granulocytes. MISTRG, NSG‐SGM3, or NOG‐EXL mice expressing these cytokines can generate not only lymphoid T and B cells but also myeloid cells such as granulocytes, macrophages, and dendritic cells (Figure 1). In sum, the development of human immune reconstitution mouse models has undergone a complex process, from the simple transplantation of PBMCs to the development of CD34^+^ HSC‐based models capable of reconstituting both innate and adaptive immune cells.^87^, ^88^, ^89^ These models involved continuous modifications to provide the necessary microenvironment for HSC differentiation. Ultimately, the transplanted CD34^+^ HSCs can reconstitute multi‐lineage immune cells, providing valuable tools for immunological research.

HLA plays a pivotal part in the education of T progenitor cells. The low efficiency of human T cell reconstitution in immunodeficient mice is believed to be related to the lack of human HLA expression in these mice^90^; however, by expressing HLA‐DR4, HLA‐A2, and other HLA molecules in immunodeficient mice, transplanted human HSCs can differentiate into CD4^+^ helper T cells, antigen‐specific CD8^+^ T cells, and functional B cells.^91^, ^92^, ^93^, ^94^

#### 5.1.2 Increasing the degree of immunodeficiency

Currently, commonly used human immune‐reconstituted mice are constructed based on mice with combined T, B, and NK cell deficiencies; however, the deficiency of T, B, and NK cells alone is not sufficient to ensure the survival of human grafts in mice, as other types of immune cells or immune molecules are still key factors influencing immune reconstitution. FLT3L, a hematopoietic growth factor, can promote the development of hematopoietic precursor cells into dendritic cells and enhance the survival and proliferation of dendritic cells. However, normal FLT3L expression in the bone marrow of immunodeficient mice can affect the effectiveness of immune reconstitution. Immunodeficient mice with the Flk2/Flt3 molecule knocked out, also known as BRGF mice, not only have impaired development of their own dendritic cells but also promote the reconstitution of human dendritic cells following transplantation of CD34^+^ HSCs, thereby improving the efficiency of human T cell and NK cell reconstitution.^95^ The binding of the macrophage‐expressed Sirpα receptor with its ligand CD47 mediates the generation of inhibitory ‘don't eat me’ signals, which inhibits the phagocytic activity of macrophages against target cells.^96^, ^97^ The Sirpα in NOD background mice exhibits structural similarity to the human form and is able to bind human CD47. As a result, introducing NOD mouse Sirpα into BRGF mice can reduce the phagocytic activity of macrophages and enhance transplantation efficiency (Figure 1). CD34^+^ HSCs transplanted into these mice can differentiate into functional immune cells without developing GVHD.^98^, ^99^, ^100^

#### 5.1.3 Reducing the occurrence of GVHD

Although GVHD after CD34^+^ HSC transplantation appears later and with milder reactions compared to PBMC transplantation, GVHD resulting from PBMC transplantation is the most common issue in immune reconstitution, severely limiting the application of such models. Different factors contribute to the occurrence of GVHD, including animal pre‐treatment methods (such as irradiation), the PBMC inoculation dose, and host immune responses. The *Prkdc* is a radiosensitive gene, and immunodeficient mice based on *Prkdc* gene mutations are commonly irradiated with low doses of γ‐rays to reduce GVHD and improve the engraftment rate of human immune cells.^101^ However, loss of *c‐kit* function impairs endogenous mouse hematopoietic stem cells, allowing engraftment of human HSC without irradiation. Thus, NSG or NOG mice with a *c‐kit* mutation (NSGW41 mice) can achieve a higher reconstitution efficiency of human immune cells after HSC transplantation free from the need of γ‐ray irradiation.^102^ In PBMC transplantation, the occurrence of GVHD resulting from the recipient mouse's MHC antigen recognition of donor T cells leads to a significantly shorter experimental window, thus impacting experimental results. Immunodeficient mice with mutations in MHC‐I and/or MHC‐II subunits (β2‐microglobulin) and H2‐Ab1 show a significant reduction in GVHD occurrence, thus extending the experimental window^59^, ^103^, ^104^ (Figure 1). Further, in TKO mice with CD47 knockout, the onset of GVHD after human PBMC transplantation is delayed.^98^

### 5.2 Improvement of the microenvironment for HSC development

#### 5.2.1 Lymph nodes

Lymph nodes are the primary sites for the residence of mature T and B cells; however, immunodeficient mice with *IL2rg* knockout have underdeveloped lymph nodes, which affects the development of transplanted human T and B cells. By specifically re‐expressing the *IL2rg* gene or thymic stromal cell‐derived lymphopoietin (TSLP) in lymphoid tissues of immunodeficient mice, the development of lymph nodes can be promoted, providing suitable developmental niches for immune cells. As a result, human T cells can reside in the lymph nodes of the mice following immune reconstitution, with a predominance of residence in the intestinal lymph nodes. In addition, they can generate antigen‐specific IgG antibodies.^105^, ^106^

#### 5.2.2 Hematopoietic niche

The bone marrow microenvironment is composed of mesenchymal stem cells (MSCs) and differentiated cells such as osteoblasts, osteoclasts, vascular endothelial cells, stromal cells, and hematopoietic cells.^107^ MSCs play a pivotal part in providing essential support for self‐renewal and differentiation of HSCs. MSCs can directly interact with HSCs through adhesion molecules on their cell membrane or regulate HSC differentiation and maturation through the secretion of various cytokines such as IL‐6, IL‐11, LIF, G‐CSF, SCF, and GM‐CSF.^108^, ^109^ In vitro co‐culture of MSCs and HSCs effectively enhances HSC proliferation, while in vivo co‐transplantation enhances HSC engraftment and differentiation, with a higher engraftment of CD13^+^ myeloid lineage cells and earlier appearance of CD19^+^ B cells.^110^, ^111^, ^112^ MSCs overexpressing PDGFB have a more pronounced regulatory effect on HSCs, promoting HSC engraftment and maintaining their self‐renewal capacity, as compared to wild‐type MSCs.^113^, ^114^ In addition, co‐infusion of MSCs and allogeneic HSCs can prevent and alleviate GVHD, facilitate HSC engraftment, and accelerate hematopoietic reconstitution.^114^, ^115^ The antioxidant N‐acetyl‐L‐cysteine (NAC) can reduce ROS levels in the bone marrow of NOD‐SCID mice, improve the bone marrow hematopoietic microenvironment, and enhance HSC engraftment efficiency. NAC‐treated NOD‐SCID mice exhibit a 10.8‐fold higher efficiency in immune reconstitution compared to untreated mice.^116^

#### 5.2.3 Organoids

Although thymus and bone marrow transplantation provide good strategies for immune reconstitution in humans, ethical issues limit the widespread application of bone marrow–liver–thymus (BLT) mice. Researchers have developed thymic organoid as a substitute for the human thymus in order to address this. In vitro cultured induced pluripotent stem cells (iPSC) can be directed to differentiate into functional thymic epithelial progenitor cells, supporting de novo construction of a T cell compartment in humanized mice engrafted with iPSC‐derived thymus organoids after CD34+ HSCs implantation.^117^, ^118^, ^119^ The transplantation of a human bone marrow MSC‐derived ossicle‐like organoid under the skin of immunodeficient mice can establish a humanized bone marrow microenvironment. The humanized bone marrow microenvironment provides a suitable homing and engraftment site for human HSCs compared to mouse bone marrow, thus promoting their differentiation and proliferation and significantly improving the engraftment efficiency of HSCs.^120^

## 6 PROSPECTS

After decades of development, researchers have successfully reconstituted the human immune system in immunodeficient mice by transplanting PBMCs or HSCs. These humanized immune‐reconstituted mice overcome interspecies differences and provide important tools for immunology‐related research; however, it is undeniable that current humanized immune‐reconstituted mice still have limitations, such as a single‐lineage immune cell repertoire, a high risk of GVHD after PBMC transplantation, and a long HSC differentiation period. Regarding the host animal, SCID mice are currently the most suitable host animal. Developing genetically engineered mice that differ from the commonly used SCID mice may be an important approach to improve the efficiency of immune reconstitution. Regarding the graft, MSC co‐transplantation with HSCs can effectively enhance the HSC differentiation capacity, and the use of organoids provides a superior microenvironment for HSC differentiation. Therefore, MSC co‐transplantation with HSCs or simultaneous transplantation of HSCs and organoids in immunodeficient mice may be important strategies for improving immune reconstitution efficiency in the future.

## AUTHOR CONTRIBUTIONS

Yixiao Bin conceived and wrote the original draft of this manuscript, Hai Zhang and Zhinan Chen revised it. All authors critically read and contributed to the manuscript, and approved its final version.

## FUNDING INFORMATION

This work was supported by Scientific and Technological Resources Coordination Project of Shaanxi Province (2020PT‐002, 2022PT‐43, 2023‐CX‐PT‐18), Special Fund for Military Laboratory Animals (SYDW_KY (2021)13) and State Key Laboratory of Holistic Integrative Management of Gastrointestinal Cancers (CBSKL2022ZZ28).

## CONFLICT OF INTEREST STATEMENT

Hai Zhang is an editorial board member of AMEM and co‐author of this article. To minimize bias, he was excluded from all editorial decision making related to the acceptance of this article for publication. The authors declare that there is no conflict of interest regarding the publication of this article.

## ETHICS STATEMENT

None.
